# Genetic Modeling of PIM Proteins in Cancer: Proviral Tagging and Cooperation with Oncogenes, Tumor Suppressor Genes, and Carcinogens

**DOI:** 10.3389/fonc.2014.00109

**Published:** 2014-05-15

**Authors:** Enara Aguirre, Oliver Renner, Maja Narlik-Grassow, Carmen Blanco-Aparicio

**Affiliations:** ^1^Biology Section, Experimental Therapeutics Programme, Spanish National Cancer Research Centre (CNIO), Madrid, Spain

**Keywords:** Pim kinases, tumors, proviral tagging, transgenics, knock-out, carcinogens

## Abstract

The PIM proteins, which were initially discovered as proviral insertion sites in Moloney-murine leukemia virus infection, are a family of highly homologous serine/threonine kinases that have been reported to be overexpressed in hematological malignancies and solid tumors. The PIM proteins have also been associated with metastasis and overall treatment responses and implicated in the regulation of apoptosis, metabolism, the cell cycle, and homing and migration, which makes these proteins interesting targets for anti-cancer drug discovery. The use of retroviral insertional mutagenesis and refined approaches such as complementation tagging has allowed the identification of *myc, pim*, and a third group of genes (including *bmi1* and *gfi1*) as complementing genes in lymphomagenesis. Moreover, mouse modeling of human cancer has provided an understanding of the molecular pathways that are involved in tumor initiation and progression at the physiological level. In particular, genetically modified mice have allowed researchers to further elucidate the role of each of the Pim isoforms in various tumor types. PIM kinases have been identified as weak oncogenes because experimental overexpression in lymphoid tissue, prostate, and liver induces tumors at a relatively low incidence and with a long latency. However, very strong synergistic tumorigenicity between Pim1/2 and c-Myc and other oncogenes has been observed in lymphoid tissues. Mouse models have also been used to study whether the inhibition of specific PIM isoforms is required to prevent carcinogen-induced sarcomas, indicating that the absence of Pim2 and Pim3 greatly reduces sarcoma growth and bone invasion; the extent of this effect is similar to that observed in the absence of all three isoforms. This review will summarize some of the animal models that have been used to understand the isoform-specific contribution of PIM kinases to tumorigenesis.

## *Pim1* as a Proviral Integration Site for Murine Leukemia Viruses

The proviral insertion site in Moloney-murine leukemia virus (PIM) proteins are a family of short-lived serine/threonine kinases that are highly conserved in multicellular organisms throughout evolution. The PIM family consists of three members, PIM1, PIM2, and PIM3. These kinases are highly homologous at the amino acid level (1) but differ partially in their tissue distribution (2). The PIM kinases have unique structural properties and are characterized by constitutive serine/threonine activity that does not depend on post-translational modifications for activation. PIM kinase activity supports the growth and survival of tumor cells *in vitro* and *in vivo* through the modification of an increasing number of shared and isoform-specific substrates, including c-myc and Histone H3, which drive transcription; eukaryotic elongation factor 4E-BP-1, which regulates translation; and Bad, which activates cell survival. Furthermore, cell cycle protein activation by PIM kinases is involved in proliferation, and PIM kinases also mediate the control of energy metabolism through the regulation of AMPK activity [reviewed in Ref. (3, 4)].

In 1984, Cuypers and co-workers identified *pim1* by cloning the retroviral integration sites in Moloney-murine leukemia virus (M-MuLV)-induced lymphomas. M-MuLV is a slow-transforming oncogenic retrovirus that produces mono- or oligoclonal tumors with a latency of several months; these tumors are usually induced by the activation or interruption of cellular genes via proviral integration.

The *pim1* gene was identified as a common insertion site in 50% of T-cell lymphomas that were induced by M-MuLV or AKR-MCF 247 virus (5). Proviral insertion also occurred with a frequency of 45% in the vicinity of *c-myc*, and an analysis of transplanted lymphomas with insertions in the *c-myc* and *pim1* loci revealed that primary lymphomas were poly- or monoclonal tumors, emphasizing the potency of cooperation between these two genes in driving tumor progression (6–8). Integrations into the *pim1* locus (mouse chromosome 17, which corresponds to human 6p21) lead to increased mRNA production, increased levels of wild-type protein, and the development of T- and B-cell lymphomas (5, 8–10). Proviral insertions (in the sense direction) into the 3′-terminal exon of the pim1 gene result in the removal of the 3′ UTR, which is responsible for reduced mRNA stability. Therefore, the loss of this region by proviral insertion results in increased Pim1 expression levels.

Integrations of Moloney-murine leukemia virus into the *pim2* locus occur at a lower frequency than integrations into the pim1 locus (8 versus 20%), but this frequency is increased in Pim1 heterozygous (10%) and homozygous (25%) knock-out (KO) mice (11). Integration into the *pim2* locus leads to enhanced mRNA production and promotes T- and B-cell lymphomas.

Several mouse strains have been used to study the proviral integration of M-MuLV; most of these studies have been carried out in the BALB/c and C57BL strains, but pim1 rearrangements were also observed in two T-cell lymphomas, one from an HRS/J mouse and one from a C58/J mouse. Both rearrangements appeared to result from ecotopic viral integration. Both proviruses were localized to the 3′ untranslated sequences of the *pim1* gene and were oriented in the same transcriptional direction as *pim1* (12), leading to the cleavage of the transcript at the polyadenylation site of the 5′ LTR. This premature polyadenylation may result in the removal of destabilizing sequences and thereby to the production of transcripts with increased stability (13). In addition to *pim1*, similar insertions into the 3′ UTR have been described for *pim2* and *N-myc*.

The inoculation of newborn BALB/c or C57BL1O mice with M-MuLV revealed insertions near the *c-myc, pim1*, or *pim2* genes in the primary lymphomas. After transplantation of the primary tumors, a significant enrichment in the frequency of insertions near *pim2* was observed; this frequency increased from 10% to over 50% in the transplanted tumors compared to the primary tumors (14).

Moreover, other viruses have also been shown to integrate into the *pim1* locus, but with a lower frequency. Indeed, the integration of the Friend murine leukemia virus (F-MuLV) into the *pim1* locus was reported to induce erythroleukemia, and integrations into the *c-myc* and *pim1* loci have been described in T-lymphoid leukemias (15). Rearrangements of these two genes are often associated with p53 gene alterations within the same tumor.

It has been demonstrated that a bcr-abl retrovirus that is pseudotyped with the Moloney helper virus (bcr-abl/M) can induce lymphoma in the thymus, although with a prolonged latency period compared to the v-abl-carrying virus, A-MuLV, which has not been shown to integrate into known protooncogenes. Because of its long latency period, it was assumed that, if bcr-abl-induced thymomagenesis was affected by retroviral insertion, proviral integration into cooperating loci should be detected (16). Indeed, the bcr-abl-induced tumors displayed recurring integration into *c-myc, pim1*, and *Mlvi1*, although at lower frequency than was reported for M-MuLV-induced tumors. Surprisingly, independent thymomas that were clearly of T-cell origin showed proviral insertion within the *Ahi1* region, which was previously thought to occur exclusively in A-MuLV/M-induced pre-B-cell lymphomas.

The Graffi murine leukemia virus is a non-defective retrovirus that induces granulocytic leukemia in BALB/c and NFS mice. To identify genes that are involved in Graffi MuLV-induced granulocytic leukemia, genetic alterations that had been previously described for other MuLV-induced leukemias were examined. Three percent of tumors generated by the intraperitoneal inoculation of newborn NFS and BALB/c mice with Graffi MuLV showed rearrangements in *c-myc* and *pim1*, indicating that cooperation between *c-myc* and *pim-1* activation may also play a role in myeloid leukemogenesis (17).

In summary, *pim1* is a very common insertion site in T-cell lymphomas that are induced by M-MLuV infection in different mouse strains. In contrast, in B-cell lymphomas that are induced by M-MLuV or other lymphoid diseases induced by different viruses, the frequency of insertions at *pim1* is greatly reduced (Table 1).

**Table 1:** **Pim1 as a proviral integration site**.

Virus	Mouse strain	Locus of insertion (frequency %)	Tumor type
M-MuLV	BALB/c, C57BL	3′Region of *pim1* (50%)	Early T-cell lymphoma (5)
M-MuLV	BALB/c, C57BL	3′Region of *pim1* (50%); 3′region of *c-myc* (45%)	Early T-cell lymphoma (6)
M-MuLV	BALB/c	3′and 5′Regions of *pim1* (50% early lymphomas, 7% late lymphomas)	Early and late T-cell lymphoma (8)
M-MuLV	BALB/c, C57BL	Oligoclonal and monoclonal in *c-myc* and *pim1*	Early T-cell lymphoma (7)
M-MuLV	AKXD	3′Region of *pim1*; 3′region of *c-myc*	T- and B-cell lymphomas (9)
Ecotropic virus	HRS/J, C58/J	3′Region of *pim1* (11%)	T-cell lymphoma (12)
Mo+, Mo + PYwt, Mo + PyF441 M-MuLVs	NIH Swiss	*Mo* + *pim1* (33%), *Mo* + *PYwt pim1* (13.3%), *Mo*+ *PyF441* no insertion	Lymphoblastic lymphoma (18)
M-MuLV	BALB/c, C57BL, B6 nude (nu/nu)	No insertion at the *pim1* locus	B-cell lymphomas (19)
Transplanted lymphomas induced by M-MuLV	BALB/c, C57BL10	Retain integration in *c-myc, pim-1* enrichment of *pim-2* insertion from 10% to over 50%	T-cell lymphoma (14)
M-MuLV	BALB/c, C57BL10	*pim2* and *pim1* (21%); 10% of cells affected	T-cell lymphoma (14)
Friend helper leukemia virus (F-MuLV)	ICFW	*p53* (35%), *p53* and *pim-1* (24%), *p53* and c*-myc* (6%), c*-myc* (12%)	Erythroleukemia (15)
M-MuLV and bcr-abl/M	BALB/c	M-MuLV: *pim1* (33%); bcr-abl/M: *pim1* (14%)	Thymoma (16)
Graffi murine leukemia viruses	Balb/c and NFS	*pim1* (3%)	Myeloblastic leukemia (17)

## Transgenic Pim Mouse Models to Study Oncogenic Cooperation

The manipulation of the mouse genome can be used to model the somatic mutations that are found in naturally occurring human cancers; the aim of these studies is to establish their etiological significance and determine the mechanisms by which they predispose to malignancy (20).

In particular, transgenic mouse models have been developed in order to address the oncogenic potential of Pim kinases. In most of these models, only *pim1* expression has been genetically altered, but there are also a few studies in which the functions of *pim1* homologs were evaluated using specific transgenic models.

van Lohuizen and co-workers generated an *Eμ-pim1* transgenic strain that contains a duplicated immunoglobulin heavy enhancer (21) upstream of the *pim1* promoter and a single murine leukemia virus (MuLV) long terminal repeat within the 3′ untranslated region. The *Eμ* enhancer achieved a high level of transcription, and the *pim1* transgene was overexpressed in lymphoid tissues at similar levels in both T- and B-cells. However, only 5–10% of these mice developed T-cell lymphoma with a latency period of 240 days (22).

*Eμ-Pim1* mice have a very low rate of developing T-cell lymphomas with a long latency, but infection with M-MuLV virus dramatically increases the incidence of lymphoma and shortens the latency of T-cell lymphoma development (56–64 versus 154 days) (22). The activation of either *c-myc* or *N-myc* was involved in every analyzed tumor, emphasizing the importance of the *pim/myc* collaboration in tumor development (22–24). Additionally, retroviral insertions in the *ice1/gfi1/pal1/evi5* locus were observed in nearly 80% of the lymphomas (25).

As mentioned above, the transgene in *Eμ-pim1* transgenic mice is expressed in both B- and T-cells. This pattern contrasts with that of *Eμ-c-myc* transgenic mice, which develop spontaneous B-cell lymphoma with a high incidence rate. Moreover, *Eμ-pim1* transgenic mice do not display anomalies in the bone marrow, whereas the bone marrow of *Eμ-c-myc* transgenic mice was profoundly abnormal, showing enhanced proliferation of premature B-cells (26, 27). This apparent lack of increased hematopoietic cell proliferation in *Eμ-pim1* transgenic mice could indicate that *pim1* alone cannot cause massive cell proliferation in any of the hematopoietic compartments in which it is expressed (22). Likewise, the retroviral infection of transgenic *Eμ-myc* mice led to the integration of the provirus in the *pim1* locus in 35% of tumors (10). Although *Eμ-c-myc* transgenic mice already displayed a high incidence of B-cell lymphomas with a short latency, the development of tumors was enhanced after retroviral infection. These results support the findings that the *myc* and *pim1* genes collaborate in lymphomagenesis.

The cooperation between the *myc* and *pim* genes was ultimately proven by experiments using *Eμ-c-myc;Eμ-pim1* and *Eμ-c-myc;Eμ-pim2* double-transgenic mice, in which pre-B-cell leukemia presented around birth (28, 29). By crossing heterozygous animals of both genotypes, it was observed that *Eμ-c-myc;Eμ-pim1* mice expressing high levels of c-myc were not viable, whereas *Eμ-c-myc;Eμ-pim1* mice expressing low levels of c-myc were viable and showed a low tumor incidence. To gain insight into the cause of the perinatal lethality of the double-transgenic mice that expressed the indicated transgenes at high levels, 17–19-day-old fetuses were collected and examined histologically. Major changes were observed in the livers and spleens of these fetuses, and immunochemistry revealed that the expanded cell population in the fetuses carried the B-cell-specific cell surface marker B220, indicating that these cells represent pre-B-cells. The transplantation of these leukemic cells into nude mice resulted in tumor outgrowth within 54 days in 80% of the animals. The authors concluded that upon transplantation into recipient mice, the embryo-derived double-transgenic leukemic cells frequently seemed to be derived from different ancestor cells; these ancestor cells progressed to highly malignant monoclonal tumors, indicating that additional selective forces may act on these cells, resulting in the outgrowth of adapted subclones of tumor cells (28).

Breuer et al. developed another *pim1* transgenic mouse line, *H_2_K-pim1*. This construct was derived from the *Eμ-pim1* construct, in which the *pim1* promoter region was replaced with that of the *H_2_K* promoter. Although 10% of *Eμ-pim1* transgenic mice developed tumors within 240 days, *H_2_K-pim1* mice did not develop tumors spontaneously, and the administration of chemical carcinogens such as *N*-ethyl-*N*-nitrosourea was needed to increase the incidence of lymphomas; this model will be described below (23).

Berns and co-workers also used proviral tagging to identify collaborating oncogenes and genes that contribute to tumor progression. Four transgenic lines (*TDK-pim1, Eμ-c-myc, H_2_K-myc*, and *Eμ-Bmi1*) were used in this study, and activation by retroviral tagging allowed the authors to assess the collaborative roles of *c-myc, N-myc, pim1, pim2, Bmi1*, and *Gfi1* (30). Pim1 transgenic mice were generated using a TDK expression cassette, in which the immunoglobulin heavy chain enhancer is combined with the *pim1* promoter and the M-MLuV terminal repeat. The only difference from the previously described *Eμ-pim1* mouse model is the FVB/N strain background. As was described before, the activation of *c-myc, N-myc*, and *Gfi1* was observed in these *TDK-pim1* transgenic mice, whereas the activation of *pim1, pim2, c-myc*, and *N-myc* was predominant in the *Bmi1* transgenic mice (30). Furthermore, the expression patterns of the transgenes in these mouse lines promoted different types of tumors, yielding almost exclusively B-cell lymphomas in the *Eμ-c-myc* transgenic mice, whereas the *H_2_K-myc* transgenic mice developed mainly T-cell lymphomas. In contrast, the *TDK-Bmi1* mice showed predisposition to both B- and T-cell lymphomas.

More recently, An et al. generated another transgenic mouse overexpressing human *pim1* using *vav* hematopoietic regulatory elements and SV40 sequences (31). *Pim-Tx* mice showed enhanced hematopoiesis characterized by an increased number of hematopoietic stem/progenitor cells (Lin-Sca-1^+^ c-Kit^+^) and cobblestone area-forming cells, along with a higher degree of BrdU incorporation in long-term hematopoietic stem cell populations and a greater ability to reconstitute lethally irradiated mice.

In addition to hematopoietic tissues, the oncogenic role of Pim1 and its synergy with c-myc in tumorigenesis have also been studied in prostatic epithelium by Wang and co-workers. In this case, lentiviral constructs that coexpressed pim1, a kinase-dead (K67M) mutant pim1, human c-myc, or c-mycCS62D were generated and used to infect prostate cell suspensions isolated from C57BL/6 mice singly or in combination (c-myc/pim-1 or c-myc/K67M) (32). After infection, these cells were combined with rat urogenital sinus mesenchyme and grafted under the renal capsules of SCID mice to regenerate prostates. The results indicated that pim1 was also weakly oncogenic in the prostate environment, but it cooperated dramatically with c-myc to induce prostate cancer within 42 days, generating large hemorrhagic tumors, while the control, pim1, K67M, or c-myc or c-myc/K67M grafts were small and did not differ significantly in their sizes. This finding was reinforced by the observed increase in the active serine-62 (S62)-phosphorylated form of c-myc in the c-myc/pim1 tumors. However, grafts expressing the phosphomimetic c-myc CS62D mutant had higher rates of proliferation than grafts expressing the wild-type c-myc but did not form tumors like the c-myc/pim1 grafts, indicating that the cooperation between pim1 and c-myc *in vivo* involves mechanisms other than the enhancement of c-myc activity by S62 phosphorylation. In contrast to pim1 grafts, all of the c-myc and c-myc/K67M grafts showed multiple foci of high-grade prostate intraepithelial neoplasia (PIN), a putative precursor lesion for prostate carcinoma, but none of them showed evidence of invasive cancer after 42 days (32).

To evaluate pim1 inhibition as a therapeutic target for prostate cancer, particularly for myc-expressing tumors, the same authors examined the molecular consequences of pim1 and myc overexpression in the prostate and the effects of depleting pim1 in prostate carcinoma cells with high levels of myc. For this purpose, the mouse prostate tissue (MPT) cell line was generated from c-myc/pim1 prostate tissue recombinant grafts that overexpressed both oncogenes. To study the effect of pim1 expression, these cells were infected with an shRNA against mouse pim1 (named shPim1#1). The knock-down of pim1 did not lead to a compensatory increase in pim2 or pim3, and there were no appreciable changes in the levels of myc protein in MPT cells. However, the knock-down of pim1 substantially reduced cellular proliferation and focus-forming ability, which are typical of cancer cells, compared with cells expressing a control shRNA (shControl#1). In addition, the *in vivo* tumorigenicity was evaluated by injecting shPim1#1 or shControl#1 MPT cells subcutaneously into athymic nude mice. No tumors were observed in the pim1 knock-down group after 84 days, whereas 60% of the mice in the control group formed large tumors. A subsequent analysis of the expression of different kinases in MPT cells showed that, although there were no consistent changes in Akt signaling, Erk1/2 phosphorylation was consistently reduced in pim1 knock-down cells compared to the corresponding control cells (although the stable overexpression of pim1 in prostate cancer cells did not alter Erk phosphorylation). In contrast, an examination of pim1-expressing recombinant prostate grafts *in vivo* showed evidence of enhanced Erk1/2 phosphorylation. Overall, these results indicated that pim1 may not be sufficient to promote the onset of invasive prostate cancer but suggested that pim1 expression may be necessary for maintaining Erk signaling and, therefore, prostate cancer cell tumorigenicity (32).

More recently, we generated a conditional Pim1 transgenic mouse model to assess the contribution of PIM1 to neoplastic initiation and progression in the prostate epithelium. Human *Pim1* was cloned into the pVL-1 vector, and a stop cassette flanked by LoxP sequences was inserted before the *hPim1* cDNA. This construct was injected into embryonic stem cells, and embryos bearing this construct were transferred into pseudo-pregnant mice. These animals were crossed with another transgenic mouse model that expressed Cre recombinase under the control of the *PSA* promoter (*PSA61-cre*); this allowed CRE expression and the subsequent transcription of *pim1* transgene primarily in prostate tissue and, upon hormone treatment, in bladder tissue. Using these *pim1*; *PSA61-cre* mice, the effect of pim1 overexpression was explored in three different settings: after hormone treatment, during aging, and in combination with the absence of one *pten* allele. We found that pim1 overexpression moderately increased the severity of mouse prostate intraepithelial neoplasia (mPIN) in all three settings. Furthermore, Pim1 overexpression in combination with hormone treatment increased inflammation in the surrounding target tissues, leading to pyelonephritis in the transgenic animals. The increased inflammation observed in the pim1 transgenic mice after hormone treatment may be due to a positive feedback loop between hormone treatment and pim1 transgene expression because only these mice developed inflammation and subsequent pyelonephritis. An analysis of the senescence induced in these prostatic lesions showed that the lesions generated in the presence of inflammation exhibited different behavior than those that were induced in the absence of inflammation. In the presence of inflammation, high-grade prostate preneoplastic lesions (mPIN grades III and IV) did not show any senescence markers and demonstrated high levels of Ki67 staining. However, untreated animals without inflammation expressed senescence markers and had low levels of Ki67 staining in similar high-grade lesions. These data suggested that pim1 may contribute to progression rather than initiation in prostate neoplasia, indicating that pim1 plays a role in promoting prostate tumorigenesis *in vivo*, although it displayed distinct oncogenic activities depending on the disease stage (33). After prolonged hormone treatment, pim1 was also expressed in the urothelial epithelium, inducing urothelial hyperplasia in the bladder and hyperplasia of the urethra in mice overexpressing pim1 alone or with the simultaneous loss of one *pten* allele. This hyperplasia may lead to light occlusion of the bladder and urethra because 50% of the animals overexpressing the pim1 transgene developed pyelonephritis and needed to be sacrificed. These data suggested that an increase in pim1 expression may contribute to the progression of urothelial neoplasia, rather than its initiation, and that urothelial hyperplasia may be an important factor in the development of pyelonephritis (34).

In addition to these pim-1 transgenic mouse models, there are a few studies in which the pim2 and pim3 isoforms were genetically modified in order to characterize the function of these proteins. There is evidence from M-MuLV viral tagging experiments suggesting that the oncogenic behavior of Pim2 is similar to that of Pim1; for example, both genes are highly expressed in mitogenically stimulated hematopoietic cells, and their transcription is induced in response to the same cytokines. To follow up on these data, Allen and co-workers developed an *Eμ*-*pim2* transgenic mouse model. In this mouse model, the overexpression of a *pim2* transgene in lymphoid cells predisposed mice to T-cell lymphomas that were similar to those promoted by *pim1* transgenes. In particular, 40% of the *Eμ-pim2* transgenic animals developed T-cell lymphomas within 1 year of age, and these lymphomas showed similar histological characteristics to those observed in *Eμ-pim1* mice. Moreover, a strong collaboration between the *Eμ*-*pim2* and *Eμ-c-myc* transgenes was observed; pre-B-cell leukemia developed in neonatal bi-transgenic animals (29). These animals developed severe leukemia and died before 28 days of age. At birth, their appearance was normal, but their growth was noticeably retarded thereafter, reaching only half the weight of their litter mates. A consistent leukemic phenotype was observed in these animals, with large undifferentiated lymphoblastoid cells infiltrating most tissues. These results confirmed that *pim2* was an oncogene that potently collaborated with *c-myc*, even when it was only modestly overexpressed and that the resulting tumors were similar to those obtained with the *pim1* transgene (29).

Wu et al. addressed the role of Pim3 in the progression of hepatocellular carcinoma (HCC) by generating a transgenic mouse that selectively overexpressed human PIM3 in the liver (35). This line was developed by cloning the full-length human *Pim3* cDNA downstream of the mouse *albumin* enhancer/promoter gene (36) and introducing this construct into fertilized oocytes of C57BL/6 mice following a standard transgenic technique. When these pim3 transgenic mouse-derived hepatocytes were analyzed, Bad was found to be constitutively phosphorylated at Ser^112^ (indicating that the overexpressed PIM3 was functional) and the levels of cyclin D1 and proliferating cell nuclear antigen were increased relative to wild-type mice. Moreover, a cell cycle analysis of hepatocytes isolated from Pim3 transgenic mice indicated that the proportion of cells in the G2/M phase was significantly increased, suggesting that PIM3 overexpression could accelerate the cell cycle progression of hepatocytes. However, the development of spontaneous HCC was not observed in liver-specific Pim3 transgenic mice (35). Nevertheless, the administration of diethylnitrosamine (DEN), a potent hepatocarcinogen, induced a higher proliferation rate in pim3 transgenic mouse cells. While HCC was detected in 40% of wild-type mice 10 months after this treatment, a higher frequency (80%) of Pim3 transgenic mice developed HCC, and those mice had a heavier burden. These observations indicated that PIM3 alone could not cause the development of HCC, but could accelerate its development induced by a hepatocarcinogen.

All of these described models are summarized in Table 2.

**Table 2:** **Mouse models overexpressing Pim proteins**.

Mouse model	Expression of transgene	Phenotype
*Eμ-pim1*	B- and T-cells	Enlargement of the spleen
		Monoclonal T-cell lymphoma with high expression of c-myc; 10% penetrance, 240 weeks latency (22–24)
*H_2_K-pim1*	B- and T-cells	No spontaneous tumors (23)
*Pim1-Tx*	Lymphoid lineage: higher expression in B lymphoid cells than in myeloid cells	Enhanced hematopoiesis, higher BrdU incorporation in long-term HSC populations, and greater ability to reconstitute lethally irradiated mice
		Acute lymphoblastic leukemia/lymphoma; 10% penetrance, 20–62 weeks latency (31)
*pim1; PSA61-Cre*	Prostate and bladder epithelium	100% of 10-month-old mice developed low-grade mPIN lesions
		The incidence of low-grade and high-grade mPIN lesions increases after two rounds of hormone treatment. All (100%) of the mice developed mPIN lesions at 24 weeks of age, with a 10% incidence of high-grade mPIN and *in situ* carcinoma (33)
		All (100%) of the mice developed high-grade bladder and urothelial hyperplasia after two rounds of hormone treatment, inducing pyelonephritis (34)
*Eμ-pim2*	B- and T-cells	T-cell lymphoma in 40% of the mice after 1 year (29)
*alb-pim3*	Hepatocytes	No tumors after 1 year (35)
Tissue recombination model coupled with lentiviral-mediated gene transfer	Prostate cells from 6-week-old C57BL/6 mice infected with lentivirus	Pim1 is weakly oncogenic in naïve adult mouse prostatic epithelium. However, it cooperates dramatically with c-myc to induce high-grade prostatic cancer with NE differentiation
		100% penetrance in 6 weeks (32)

## Pim Knock-Out Mice

To further determine the role of Pim1 in tumorigenesis, Pim KO mice have been widely used. In general, none of the individual Pim-KO models (with depletion of one of the three Pim kinases) developed a notable phenotype. These mice do not display any overt abnormalities; all of the major organs were within the normal range of size and structure as indicated by morphological and histological analyses. The behavior and body weight of the animals were also normal for all genotypes (homozygous knock-outs and heterozygous littermates) (37).

Over the years, only mild hematopoietic impairments have been detected in the single Pim-KO models, which is surprising given the high evolutionary conservation of these kinases.

In 1993, two studies involving Pim1-KO mice were published. Laird and co-workers generated mice that carried a *pim1* allele incapable of producing a functional Pim1 protein. These mice were generated by deleting the promoter sequences, transcription and translation initiation sites and a large segment of the coding region including the conserved lysine residue of the ATP binding site of the protein kinase domain (38). The resulting Pim1-deficient mice are ostensibly normal, healthy, and fertile. The only reported phenotype was a significantly smaller erythrocyte mean cell volume (MCV) compared to wild-type littermates (37). Because the peripheral blood concentration of erythrocytes was not increased in Pim1-deficient mice, hemoglobin levels were reduced. Further rescue experiments using a Pim1 transgene with an expression level similar to the endogenous *pim1* gene showed that MCV levels could be restored to just below the levels of wild-type littermates (37).

Another study, published by Domen and co-workers, analyzed the early B lymphoid compartment from both Pim1 null mutant and *Eμ-pim1* transgenic mice. These null mutant mice also lacked an obvious phenotype but showed an impaired response to interleukin 7 (IL-7) and steel factor (SF). A comparison of the total number of bone marrow cells recovered from Pim1 mutants and their wild-type controls revealed no significant differences. The level of Pim1 expression appears to be a determining factor in the ability of these cells to respond to IL-7 and SF. The impaired response in null mutant mice could be rescued by the introduction of a functional *pim1* transgene. Moreover, the overexpression of Pim1 facilitated the derivation of primitive lymphoid cell lines that are dependent on combined stimulation with IL-7 and SF or insulin-like growth factor 1 (39). However, the absence of Pim1 in null mutant mice did not lead to a total lack of responsiveness to IL-7 and SF, which indicates that Pim1 is more of a modulator than a mediator for these factors (39).

The animal model used in the previous study was later examined by Konietzko and colleagues, who determined Pim kinase expression after long-term potentiation (LTP) stimulation. In the brain, Pim1 is induced by plasticity-producing stimulation and is instrumental in the formation of enduring LTP. Pim1 is also a determining factor for establishing long-lasting changes in synaptic strength (40). Pim1-deficient mice showed normal synaptic transmission and short-term plasticity. However, they failed to consolidate enduring LTP, even though Pim2 and Pim3 are constitutively expressed in the hippocampus and Pim3 expression is similarly induced by synaptic activity (40). This impairment in LTP consolidation may affect certain forms of long-term memory because synaptic plasticity appears to play a role in several physiological and pathological processes of the adult brain such as learning and memory (41, 42). Overall these findings may indicate that Pim1 plays an important role in regulating the functional changes that underlie long-term synaptic plasticity (40).

All of the mice that were deficient in only one Pim kinase (single-KO) displayed mild phenotypes with only slight impairments. However, this is different from animals lacking all three Pim kinases. Although mice lacking expression of Pim1, Pim2, and Pim3 [referred to as triple knock-out mice (TKO)] are viable and fertile, they showed a profound reduction in body size at birth and throughout postnatal life. This reduction is due to a decrease in cell number rather than a decrease in cell size (43). Similar to the Pim1 single-KO mice, TKO mice displayed a decreased MCV in erythrocytes. They also have a decreased splenic B-cell fraction in addition to other B-cell impairments such as a decrease in the IL-7-mediated proliferation of late pre-B-cells. In addition, the *in vitro* response of distinct hematopoietic cell populations to growth factors is severely impaired. For example, bone marrow cells showed an impaired growth response to IL-3. In particular, the Pim proteins are required for the efficient proliferation of peripheral T lymphocytes mediated by synergistic T-cell receptor (TCR) and interleukin-2 signaling (43).

Because PIM kinases are attractive targets for anti-cancer drug discovery, it is important to further determine the relative contribution of the different isoforms to tumorigenesis *in vivo* and to understand how their individual inhibition might affect tumor growth and the normal physiology of organisms.

Using the TKO mice generated by Mikkers et al., we explored whether the inhibition of specific isoforms is required to prevent the sarcomas induced by treatment with the carcinogen 3-methylcholanthrene (44). We showed that the absence of Pim2 and Pim3 greatly reduced sarcoma growth and that the extent of this reduction was similar to that observed in the absence of all three isoforms. This model of sarcoma generally involves bone invasion by the tumor cells. The lack of Pim2 and Pim3 reduced tumor-induced bone invasion by 70%, and this reduction is comparable to the reduction of tumor-induced bone invasion in the absence of all three isoforms (44), although the absence of all three isoforms is necessary to achieve the maximum effect. These data concur with data derived from cell assays using mouse embryonic fibroblasts showing reduced proliferation rates and resistance to oncogenic transformation.

An et al., using the Pim-TKO mice that were generated by Mikkers et al., were able to show that hematopoietic stem cells (HSCs) from Pim1-KO mice had an impaired long-term hematopoietic repopulating capacity in secondary and competitive transplantations, whereas the data from Pim2 KO and Pim3 KO mice were similar to those from wild-type mice (31). Pim1-deficient and TKO mice had a lower peripheral blood platelet count and exhibited erythrocyte hypochromic microcytosis. The bone marrow cells of Pim1-KO and TKO mice demonstrated a decreased hematopoietic progenitor colony-forming ability. Importantly, the bone marrow cells from Pim1-KO and TKO mice also showed a significantly impaired ability to rescue lethally irradiated mice and reconstitute hematopoiesis in primary, secondary, and competitive transplant models. Furthermore, *in vivo* BrdU incorporation in long-term HSCs was reduced in Pim1-deficient and TKO mice (31, 45). Finally, cultured HSCs from Pim1-KO mice showed a reduced proliferation rate, as measured by Ki67 staining, and a higher rate of apoptosis via caspase 3 activation (31). Most interestingly, a genetic survey revealed that several genes, including Lef-1 and Pax-5, are affected by the deletion of Pim1 kinase in HSCs, emphasizing the important role of Pim1 in the function and regulation of HSCs (31).

A summary of the different Pim-KO mice and their phenotypes is shown in Table 3.

**Table 3:** **Mouse models with deletions of the Pim proteins**.

Mouse model	Phenotype	Reference
*Pim1*^−^*^/^*^−^	Erythrocyte microcytosis. Impaired response to IL7 and SF. HSCs showed impaired long-term hematopoietic repopulating capacity in secondary and competitive transplantations. Fail to consolidate enduring long-term potentiation	(31, 37, 39, 40)
*Pim2*^−^*^/^*^−^	HSCs mice behaved normally in a long-term hematopoietic repopulating capacity in secondary and competitive transplantations	(31)
*Pim3*^−^*^/^*^−^	HSCs mice behaved normally in a long-term hematopoietic repopulating capacity in secondary and competitive transplantations	(31)
*Pim1*^−^*^/^*^−^*; Pim2*^−^*^/^*^−^*; Pim3*^−^*^/^*^−^*(TKO)*	TKO mice exhibited reduced body size, a severely impaired *in vitro* response of distinct hematopoietic cell populations to growth factors, thrombocytopenia, and hypochromic erythrocytes. HSCs showed impaired long-term hematopoietic repopulating capacity in secondary and competitive transplantations	(43, 45)
*Pim2*^−^*^/^*^−^*; Pim3*^−^*^/^*^−^*(DKO) TKO*	Absence of Pim2 and Pim3 greatly reduced the sarcoma growth induced by 3MC, to an extent similar that observed in the absence of all three isoforms. The lack of Pim2 and Pim3 reduced tumor-induced bone invasion by 70%, which is comparable to the reduction of tumor-induced bone invasion in the absence of all three isoforms	(44)

## Cooperation between Other Oncogenes and *Pim*1 by Provirus Insertion or pim1 Overexpression

As has been described before, proviruses in several different tumor models are integrated into a single tumor cell clone in two or more different loci. For example, in M-MuLV-infected *Eμ-pim1* transgenic mice, lymphomagenesis is accelerated, and retroviral insertion into *c-myc* or the *N-myc* is detected with a high frequency. These data confirm that pim and myc are complementary genes in lymphomagenesis (22).

Transgenic mice that overexpress the *c-myc* gene under the immunoglobulin heavy chain enhancer (*Eμ-c-myc* mice) develop B-cell lymphomas at a high frequency (10, 27, 46). The fact that the tumors are clonal and often appear after an extended latency period suggests that additional events are necessary for their development. To identify genes that synergize with the *c-myc* transgene, *Eμ-myc* mice were infected with M-MuLV shortly after birth. As anticipated, the *pim1* gene was activated by proviral integration in 35% of the tumors, and sporadic insertions into the *pim2* and the *ahi1* loci were detected. In addition to *pim1*, three other main proviral integration sites were identified: *bmi1, bla1*, and *pal1* (10, 47). The generation of an *Eμ-Bmi1* mouse line confirmed the role of *bmi1* in lymphomagenesis; these mice had perturbed lymphoid development and were highly susceptible to B- and T-cell lymphomagenesis. Proviral tagging in *Eμ-Bmi1* transgenic mice was used to identify genes that cooperate with *bmi1* in lymphomagenesis (48). The activation of the *pim* and *myc* genes was frequently observed and led to accelerated development of B- and T-cell lymphomas.

The substitution of *Eμ-c-myc* with *Eμ-N-myc* or *Eμ-L-myc* also resulted in enhanced lymphomagenesis (49). Complementation of the *Eμ-N-myc* and *Eμ-L-myc* transgenic mice by breeding them with *Eμ-pim1* animals led to the more rapid development of lymphoid malignancies and a dramatically higher incidence, but the lineage specificity prescribed by the *Eμ-N-myc* and *Eμ-L-myc* transgenes was maintained (B- and T-cells, respectively). *Eμ-N-myc;Eμ-pim1* bi-transgenic mice had severe anemia and lymphoma with involvement of all lymphoid organs, while a profound acceleration of lymphomagenesis was observed in *Eμ-L-myc;Eμ-pim1* bi-transgenic mice. Large immature lymphoblasts were detected in blood smears of double-transgenic animals, demonstrating a high degree of leukemia. In addition, the different oncogenic potential of the *myc* genes was revealed by the average latency period of tumor manifestation; the latency period was 36 days for *Eμ-N-myc:Eμ-pim1* mice and 94 days for *Eμ-L-myc;Eμ-pim1* mice, but *Eμ-c-myc;Eμ-pim1* animals developed pre-B-Cell leukemia prenatally (49).

In 1997, it was shown that the *eis-1/pal-1/gfi-1/evi-5* locus serves as a target for M-MuLV proviral insertions in pre-B-cell lymphomas in *Eμ-myc* transgenic mice (20%) and in T-cell lymphomas in H_2_K-myc (75%) and *Eμ-pim1* (93%) transgenic mice (25). This common insertion site had been identified as a region containing several independent integration clusters: *eis1, gfi1*, and *evi5*. Proviral insertion into the different integration clusters upregulates the transcription of the *gfi1* gene, which is located in the *pal-1* locus. Transgenic mice that constitutively expressed high levels of *gfi1* in the T-cell lineage were later generated using the proximal *lck* promoter (*lck-gfi1*). These animals appeared to have unexpectedly low thymocyte numbers and to be weakly predisposed for lymphomagenesis. However, the coexpression of a transcriptionally deregulated *pim1* gene restored the number of mature T-cells in the lymph nodes and spleen in double-transgenic mice. In addition, after a latency period of 114 days, almost all of the double *lck-gfi1*; *Eμ-pim1* transgenic mice developed T-cell lymphoma, which demonstrated the potential of *gfi1* as a dominant oncogene and its ability to synergize with *pim* gene. A similar accelerated rate of lymphoma development was observed when *lck-gfi1* mice were crossed with mice that carried an *L-myc* gene that was targeted to be expressed at high levels in T-cells. In this case, the mean latency period for tumor development was 148 days, again providing direct evidence for a cooperative effect between *myc* and *gfi1* (50).

On the other hand, retroviral (M-MuLV) insertion in transgenic mice expressing Myc under the T-cell-specific CD2 locus control region (*CD2-myc*) led to the identification of the *til1* locus; insertions into *til1* result in the increased transcription of the *runx2/cbfa1/aml3/pebp2αA* gene (51–53). Runx2 is one member of the core binding factor family, a set of heterodimeric regulatory proteins with vital roles in hematopoiesis and osteogenesis. It has been reported that T-cell development is perturbed when the *runx2* oncogene is overexpressed; this change leads to the development of spontaneous lymphomas at a low frequency and acts synergistically with myc (54). Retroviral infection of *CD2-runx2* transgenic mice identified *c-myc* and *pim1* as collaborating genes (55). Moreover, insertional mutagenesis (M-MuLV) of *CD2-myc;CD2-runx2* double-transgenic mice also led to insertions in the *pim1* locus (51). To assess the possible functional redundancy between the *runx2* oncogene and known myc-collaborating genes in T-cell lymphomagenesis, mice overexpressing runx2 (*CD2-runx2*) were crossed with mice carrying the Pim1 transgene (*Eμ-pim1*). *CD2-runx2*; *Eμ-pim1* animals displayed a significant increase in tumor onset compared to either *CD2-runx2* or *Eμ-pim1* littermate controls. At 250 days of age, 66% of the double-transgenic mice succumbed to spontaneous T-cell lymphoma, whereas only 23% of the *CD2-runx2* mice and none of the *Eμ-pim1* mice did. This work demonstrated that in addition to the strong synergy between *runx2* and *myc, runx2* seemed to collaborate independently with *pim1*, demonstrating that it contributed to T-cell lymphoma development in a unique manner with a dominant effect on the tumor cell lineage and phenotype (55).

Retroviral insertion was also used to elucidate the role of *pim1* in large B-cell lymphomas. Mutations and chromosomal rearrangements involving the *bcl6* gene have been found in the majority of diffuse large B-cell lymphomas in humans (56). An inducible transgenic mouse model that is doubly transgenic for the tetracycline-responsive *tet-o-bcl6* gene and the tissue-specific tetracycline-transactivating protein *EμSR-tTA* has been constructed to investigate the role of Bcl6 in this type of cancer in detail (57). By retroviral insertion (MOL4070LTR), *pim1* was identified as the most frequently recurring cooperating gene in this system, and elevated levels of *pim1* mRNA and protein expression were observed in these neoplasms (B- and T-cell type) (58).

Although the role of *pim1* in retrovirus-induced lymphomagenesis has been established, the downstream targets of Pim1 that are involved have been largely unknown. Viral infection of KO mice can be used to address whether a given gene can contribute to the development of a certain type of cancer.

In Pim1-KO mice, integration of M-MulV into the *pim2* locus, which occurs in 25% of all integrations, provokes T- and B-cell lymphoma via enhanced mRNA production (11). A further 28% of the integrations occurred in the *c-myc* and *N-myc* loci of these mice, and integration into these loci occurred more often in Pim1 heterozygous KO mice (51%), or in Pim1-wild-type mice (44%). Taking advantage of the strong interaction between *pim1* and *c-myc*, a retroviral screening approach was established to search for the retroviral activation of genes that act downstream of or in parallel to *pim1*. The frequency of integrations into the *pim2* locus was significantly higher in the *Eμ-myc*-mouse model and higher in combination with deletions of the *pim1* allele. In *Eμ-c-myc;Pim1*^−/−^ mice, more than 80% of the retrovirally induced tumors contained a proviral integration into *pim2*. Furthermore, *Eμ-myc* transgenic mice lacking the expression of *pim1* and *pim2* have been infected with M-MuLV and analyzed by a high-throughput genetic screen (59). In addition to the expected *pim3*, several other genes could be identified, including the tyrosine kinase receptor Kit and the cell cycle regulator Ccnd2. Although the exact roles of these factors in Pim signaling have yet to be elucidated, the diverse nature of these proteins suggests that Pim kinases are embedded in a complex network.

The *Eμ*-transgene system was widely used to search for genes that cooperate with various oncogenes. The *v-abl* gene is known to be implicated in several types of lymphoid neoplasms. Haupt and co-workers generated *Eμ-v-abl 40* transgenic mice and reported that these animals spontaneously develop plasmacytomas. While retroviral infection with M-MuLV only moderately accelerated tumorigenesis, the tumor type was altered: nearly all of the detected tumors after M-MuLV infection were T-cell lymphomas (60). Insertions in the *c-myc, N-myc*, and *pim1* loci were observed, indicating that each of these genes can collaborate with *v-abl* in lymphomagenesis. Furthermore, one monoclonal tumor involved both *c-myc* and *pim1* together with *v-abl*, suggesting that all three genes may collaborate.

Shinto et al. also used infection with M-MuLV to investigate the contribution of additional genes to bcl2-driven tumorigenesis (61). *Eμ-bcl2* transgenic mice sporadically develop B- or T-cell lymphoma after a long latency period. While M-MuLV mainly induced mature T-cell lymphomas in wild-type mice, infection of transgenic mice led to the development of clonal pre-B-, B-, and mainly immature T-cell lymphoma. Proviral insertion was detected in the following loci: *pim1* (6%), *pim2* (23%), and *c-myc* (26%). Some tumors showed proviral insertions close both to the *c-myc* and *pim2* genes.

The clinical outcome of lymphoma was also evaluated by Wendel et al. after generating double-transgenic mouse models. In this case, the *Eμ-c-myc* model of aggressive lymphoma (46) was adapted to the transplantation approach using retrovirally transduced hematopoietic progenitor cells (62). This transgenic construct enabled murine pim2 or constitutively active myristoylated AKT expression in the recipient C57Bl/6 mice. Therefore, when the animals were monitored for lymphomas, *Eμ-myc/pim2-* and *Eμ-myc/AKT*-expressing tumors had an accelerated disease onset compared to controls and histopathology and surface markers that were indistinguishable from those of aggressive pre-B-cell lymphomas. Hence, Pim2 and AKT activate protein translation and promote lymphomagenesis in a mouse model of aggressive and indolent lymphoma (63). Moreover, mice developing *Eμ-myc/pim2* and *Eμ-myc/AKT* tumors revealed early relapse and shortened survival when a treatment of doxorubicin was administered, and rapamycin treatment had little effect on any of the tumors. However, the combination of rapamycin and doxorubicin caused dramatic responses in the AKT-expressing lymphomas but had no effect on pim2-expressing tumors. The chemoresistance caused by AKT, but not by pim2, was therefore readily reversed by mTORC1 inhibition. Interestingly, both pim2- and AKT-expressing lymphomas relied on cap-dependent translation, and the rate-limiting factor for this translation is the activation of the cap-binding protein by phosphorylation of its inhibitor 4E-BP1, which can be further enhanced by direct eIF4E phosphorylation. The administration of silvestrol, an inhibitor of eIF4E, was able to reverse pim2-mediated rapamycin resistance in human lymphoma cells and *in vivo* (63).

In pre-B-cell acute lymphoblastic leukemia in children, chromosomal translocations can lead to the fusion of the *E2a* and *Pbx1* genes. Dedera and co-workers generated a transgenic mouse model that expresses the *E2a-pbx1* oncogene under the control of the immunoglobulin heavy chain enhancer *Eμ*. These mice developed lethal, high-grade T-cell lymphomas by 5 months of age (64). However, the disruption of thymocyte differentiation and growth control was not sufficient for malignant transformation, and additional oncogenes may be implicated in this process (64). The high latency of the clonal tumors indicates that the *E2a-pbx1* oncogene is not sufficient for malignant transformation. An M-MuLV retroviral insertion strategy was designed to identify additional genes that were able to decrease the latency of thymic lymphoma development (65). Neonatal infection substantially reduced the survival rate due to accelerated T-cell lymphoma development (81 versus 130 days). The *pim1* gene was targeted by retroviral insertions in 48% of the accelerated lymphomas, whereas <5% of the lymphomas contained activated *c-myc*, and none contained activated *pim2*. In the same study, cooperation between c-myc and pim1 was demonstrated in double-transgenic mice. The characterization of the developed tumors showed them to be monoclonal, providing some evidence that additional genetic events were required for transformation. However, although the ability of E2a-Pbx1 to cooperate with Pim1 in lymphomagenesis appears to function analogously to the interaction between Pim1 and c-Myc, this synergy does not appear to be as potent as the latter one (65).

Recently, attempts have been made to modify retroviral insertion strategies in order to search for genes that might be implicated in the later stages of tumor development. One approach is the transplantation of primary tumors into syngenic recipient mice. By the propagation of tumors induced by M-MuLV in *Eμ-pim1* or *H_2_K-myc* transgenic mice, *frat1* was identified as a collaborating gene in later stages of lymphomagenesis (66). Cell lines with high expression of Myc and Pim1 had an additional growth advantage when infected with Frat1-expressing retrovirus and transplanted. In a subsequent experiment, the authors examined the effect of enhanced Frat1 expression in transgenic mice (*Eμ-pp-frat1*) (67). As expected, Frat1 expression did not predispose the animals to spontaneous tumor development but did increase the susceptibility of these animals to M-MuLV-induced T-cell lymphomagenesis. When the *Eμ-pim1* transgene was simultaneously introduced to this mouse model, the incidence of spontaneously occurring lymphomas was significantly higher than in *Eμ-pim1* single-transgenic mice of the same genetic background. These results demonstrated that *frat1* and *pim1* collaborate in lymphomagenesis (67).

Knock-out-mice have also been successfully combined with retroviral infection models to study the collaborators of tumor suppressor genes. Mice lacking a functional *p53* gene developed tumors significantly faster after infection with M-MuLV than uninfected p53^−/−^ mice or virus-infected p53^+/+^ littermates (68). However, the degree of synergy between M-MLuV and the p53 null genotype was weaker than the synergies between either of these and the *c-myc* transgene. A similar phenotype range of T-cell tumors was observed in all p53 genotype groups, including p53^−/−^ mice, which developed thymic lymphomas as the most common of several neoplastic diseases. A lack of p53 was associated with higher rates of metastasis and the rapid establishment of tumors in tissue culture. An analysis of *c-myc, pim1*, and *pal1* demonstrated that these loci were occupied by proviruses at similar frequencies in p53 wild-type and null mice. Therefore, virus-induced tumors generally occur without the loss of p53, while the inactivation of p53 in the germ line predisposes mice to tumors that are phenotypically similar to the M-MuLV-induced ones.

Another example of the use of double-transgenic mouse models to study the function of Pim1 in T-cell development was established by crossing a *pim1* transgene into mice that are deficient in either the cytokine or TCR signal transduction pathways (69). β-selection is the process of differentiation and proliferation initiated in thymocytes that is lacking in recombination-deficient SCID or Rag-deficient mice, resulting in a differentiation block at the CD4^−^8^−^25^+^44^−^stage of α/β T-cell development (70–72). When these Rag-deficient mice were infected with M-MuLV, thymic lymphomas developed at a very high incidence and with an average latency period of 150 days. These data indicated that the M-MuLV-infected and transformed pro-T-cells of Rag-deficient mice induce lymphomagenesis, compensating for the lack of a pre-TCR signal in Rag-deficient mice (69).

Because the IL-7-IL-7R complex is critical in controlling the cellularity of the pro-T-cell compartment, *Eμ-pim1* transgenic mice were crossed to γc-deficient and IL-7-deficient mice. The results indicated that pim1 was capable of restoring thymus cellularity to an appreciable extent and that pim1 could compensate for the lack of cytokine signaling, allowing pim1 transgenic, γc-deficient or IL-7-deficient thymocytes to expand. In the same study, the question of whether the frequent proviral insertions into the *pim1* locus of CD4^+^8^+^ tumors were casually involved in the differentiation into pre-T-cell-like tumors was addressed by introducing the *Eμ-pim1* transgene into Rag-deficient mice. The thymocytes of the transgenic *Eμ-pim1;Rag*^−/−^ animals showed differentiation and slow expansion from large CD4^−^8^−^CD25^+^ into small resting large CD4^+^8^+^CD25^−^ pre-T-cells; this pim1-mediated differentiation was age-dependent. Strikingly, when the *Eμ-pim1* transgene was introduced into the CD3^−/−^ background, in which most thymocytes are blocked at the CD4^−^8^−^CD25^+^ stage, no further differentiation or expansion of pim1 transgenic CD3γ-deficient pro-T-cells was observed, regardless of age (69).

**Figure 1:**
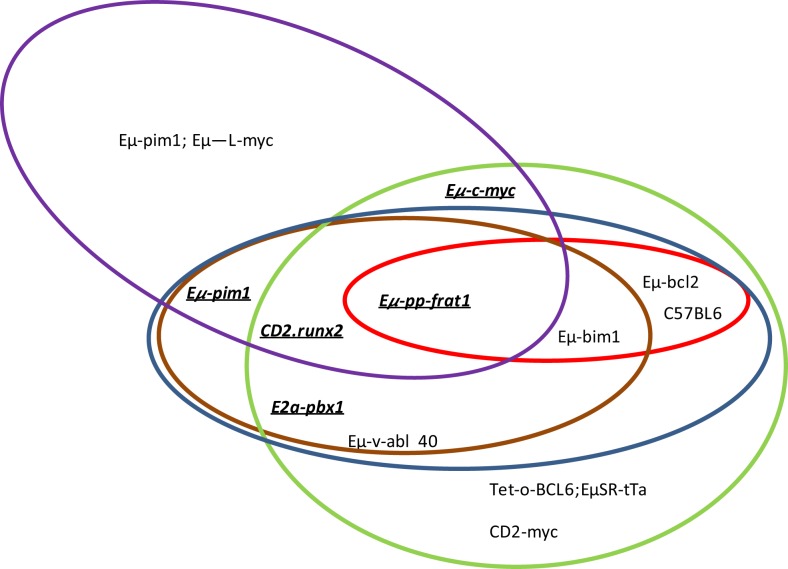
**Which genes and proviral insertion sites cooperate with Pim1?** In different mouse models, *pim1* (green ellipse), *pim2* (red ellipse), *c-myc* (blue ellipse), *N-myc* (brown ellipse) and Pal1/gfl1 (purple ellipse) are the more frequent proviral insertion sites in M-MuLV infection. The cooperation between Pim1 and some of these other genes has also been confirmed by bitransgenic mouse models (underlined and bold).

In summary, proviral tagging and bi-transgenic mouse models have allowed the identification of genes that are implicated in T-cell development and the description of cooperating genes implicated in the development of different hematological tumors. Pim has emerged as a crucial player in both processes (Tables 4 and 5; Figure 1).

**Table 4:** **Proviral integration in the pim loci and cooperating oncogenes after M-MuLV infection of transgenic mice**.

Transgenic model	Integration site	Phenotype and latency (non-infected versus infected with M-MuLV)
*Eμ-pim1*	c-*myc* (81%)	T-cell lymphomas
	N-*myc* (19%)	From 10 to 92% penetrance
	*pal1* (93%)	From 22 to 7–8 weeks average latency (22, 25, 73)
	*frat1* (17%) (transplanted infected tumors)	
*Pim1*^−^*^/^*^−a^	*pim2* (25%)	T-cell lymphomas with 100% penetrance
	c-*myc* or N-*myc* (28%)	From 14 to 18 weeks average latency (11)
*Eμ-c-myc*	*pim1* (31%)	B-cell lymphomas
	*bmi1* (35%)	From 80 to 100% incidence
	*pal1* (28%)	From 18 to 7 weeks average latency (10)
	*bla1* (14%)	
*Eμ-c-myc*; *Pim1*^−^*^/^*^−^	*pim2* (80%)	B-cell lymphoma
		No differences in penetrance (100%) or latency (50 days) (11)
*Eμ-c-myc; Pim2*^−^*^/^*^−^	*pim1* (40%)	Mature B-cell lymphoma
		No differences in penetrance or latency (30)
*Eμ-c-myc; Pim1*^−^*^/^*^−^*;Pim2*^−^*^/^*^−^	*pim3* (24%)	Mature B-cell lymphoma
	*Tpl2* (18.4%)	From 21 weeks of latency to 24 weeks of latency (30, 59)
	*cyclin D-2* (13%)	
	*c-kit* (8%)	
	*Dkmi1* (5%)	
	*Dkmi9* (8%)	
	*Dkmi11* (5%)	
	*Dkmi15* (10.5%)	
	*Dkmi20* (8%)	
	*Dkmi28* (10.5%)	
*Eμ-L-myc; Eμ-pim1*^b^	*pal1/gfi1* (37–75%)	T-cell lymphomas with 100% penetrance
	*Tiam1* (12%)	From 14 to 8.5 weeks average latency (50, 73)
	*bla1* (8.5%)	
*CD2-myc*	*pim1* (4–7.7%)	Lymphoblastic thymic lymphomas
	*til1* (32%)	From 3–33 to 100% penetrance
	*ahi1* (2.5%)	From 4 to 1 months average latency (51 –53)
*Eμ-v-abl 40*	c-*myc* (21%)	From plastocytomas to disseminated lymphoma
	N-*myc* (21%)	From 60 to 100% penetrance
	*pim1* + c-*myc* or N-*myc* (14%)	From 10 to 7 weeks average latency (60)
*Eμ-bcl2*	c-*myc* (26%)	From pre-B-cell lymphoma and plastocytoma to clonal pre-B-, B-, and immature T-lymphoma
	*pim1* (6%)	From 3–15 to 100% penetrance
	*pim2* (23%)	From up to 53 to 19 weeks average latency (61, 74)
*Eμ-Bmi1*	*N or c-myc* (39%)	B- and T-cell lymphoma with 100% penetrance
	*pim1 or 2* (31%)	From 18–25 to 9 weeks average latency (48)
*Tet-o-BCL6; EμSR-tT*^a–c^	*pim1* (21%)	From splenic B-cell lymphomas to T- and B-cell lymphomas
		From 6 to 45.3% penetrance
		From 26 to 11.5 weeks average latency (57, 58)
*E2a-pbx1*	*pim1* (48%)	Diffuse high-grade T-cell lymphomas with 100% penetrance
	*pim2* (0%)	From 18 to 11.5 weeks average latency (65)
	c-*myc* or *N-myc* (5%)	
*CD2-runx2*	c-*myc* and/or *N-myc* (82%)	Thymic lymphoma
	*pim1* (21%)	From 6 to 100% penetrance
	*pal1* (39–44%)	From 53 to 14 weeks (55)
	Increased levels of Pim1 w/o proviral integration in the *pim-1* locus	
*Eμ-pp-frat1*	c-*myc* (37–50%)	From no tumors to 100% development of T-cell lymphoma between 11 and 14 weeks (67)
	*N-myc* (6%)	
	*pim1*/*pim2* (23%)	
	*gif1/pal1* (18%)	
*Rag2*^−^*^/^*^−^	*pim1* (50%)	From no tumors to 80% lymphomas with a latency of 21 weeks (69)
	*pim2* (0%)	
*p53*^−^*^/^*^−^	*pim1* (25%) only in *p53^+^*^/−^	Thymomas with 100% penetrance
		From 26 to 13 weeks average latency (68)

*^a^In this case, the comparison is carried out between pim1-expressing and infected pim-null mice*.

*^b^Infection with wild-type and sup-F M-MuLV*.

*^c^Infection with MOL4070LTR*.

**Table 5:** **Contribution of Pim1 overexpression to tumorigenesis in double-transgenic mouse models**.

Mouse model	Single-transgenic phenotype	Double-transgenic phenotype
*E2a-Pbx1*; *H_2_K-pim1*	E2a-Pbx1: 13% incidence lymphoma at 24 weeks H_2_K-pim1: no tumors in 1 year	Lethargy, respiratory distress, and abdominal distension due to aggressive lymphomas, 100% incidence at 13 weeks (65)
*Eμ-c-myc*;*Eμ-pim1*	*EμPim1*: enlargement of the spleen	Pre-B-cell leukemia in uterus; 100% incidence (28)
	Monoclonal T-cell lymphoma; 10% incidence at 34 weeks of age	
	*Eμ-c-myc*: pre-B-cell lymphomas; 75% incidence latency between 10 and 53 weeks of age	
*Eμ-L-myc*;*Eμ-pim1*	*Eμ-pim1*: enlargement of the spleen	T-cell lymphomas; 80% incidence at 12 weeks (49)
	Monoclonal T-cell lymphoma; 10% incidence at 34 weeks of age	
	*Eμ-L-myc*: T-, B-, and pre-B-lymphomas; 8% incidence at 53 weeks of age	
*Eμ-N-myc*;*Eμ-pim1*	*Eμ-pim1*: enlargement of the spleen	B- and pre-B-lymphomas; 95% incidence at 5 weeks of age (49)
	Monoclonal T-cell lymphoma; 10% incidence at 34 weeks of age	
	*Eμ-N-myc*: B- and pre-B-lymphomas; 50% incidence between 13 and 16 weeks of age	
*Lck-gfi-1*; *Eμ-pim1*	*Lck-gfi-1*: developmental block of early T-cell development leading to a loss of thymic cellularity. T-cell lymphomas; 15% incidence at 28.5 weeks of age	Thymus cellularity restored. T-cell lymphomas; 82% incidence at 16.2 weeks of age (50, 75)
	*Eμ-pim1*: enlargement of the spleen	
	Monoclonal T-cell lymphoma; 10% incidence at 34 weeks of age	
*CD2-runx2*;*Eμ-pim1*	*CD2-Runx2*: T-cell lymphomas; 23% incidence at 53 weeks	T-cell lymphoma; 66% incidence at 36 weeks (55)
	*Eμ-Pim1*: enlargement of the spleen	
	Monoclonal T-cell lymphoma; 10% incidence at 34 weeks of age	
*CD2-runx2*;*Eμ-pim1; CD2-c-myc*	*CD2-Runx2*: T-cell lymphomas; 23% incidence at 53 weeks	T-cell lymphoma; 100% at 5 weeks (55)
	*Eμ-pim1*: enlargement of the spleen	
	Monoclonal T-cell lymphoma; 10% incidence at 34 weeks of age	
	*CD2-c-myc*: T-cell lymphomas; 100% incidence at 7 weeks	
*Eμ-pim1; γ_c_*^−^*^/^*^−^	*Eμpim1*: enlargement of the spleen	Thymus cellularity restored (69)
*Eμ-pim1*;*IL17*^−^*^/^*^−^	Monoclonal T-cell lymphoma; 10% incidence at 34 weeks of age	
	*γ*_c_^−^*^/^*^−^and *IL17*^−^*^/^*^−^: significant reduction in thymocyte number	
*Eμ-pim1;Eμ-pp-Frat1*	*Eμpim1*: T-cell lymphoma; 12% incidence at 26 weeks of age	T-cell lymphoma; 50% incidence at 26 weeks. High levels of expression of *c-myc* (67)
	*Eμ-pp-Frat1*: no tumors at 26 weeks of age	
*Eμ-pim1*;*Rag3*^−^*^/^*^−^	*Eμpim1*: enlargement of the spleen	Thymus cellularity restored in an age-dependent manner in *Rag3*^−^*^/^*^−^ but not *CD3γ*^−^*^/^*^−^mice (69)
*Eμ-pim1*; *CD3γ*^−^*^/^*^−^	Monoclonal T-cell lymphoma; 10% incidence at 34 weeks of age	
	*Rag3*^−^*^/^*^−^ and *CD3γ*^−^*^/^*^−^; differentiation block at the CD4^−^8^−^25^+^44^−^ stage of αβ T-cell development	
*Eμ-c-myc*;*Eμ-pim2*	*Eμpim2*: T-cell lymphoma; 40% incidence after 53 weeks	Severe leukemia, some harboring simultaneous T-cell lymphoma; 100% incidence at 3–4 weeks of age (29)
	*Eμ-c-myc*: Pre-B-cell lymphomas; 75% incidence and latency between 10 and 53 weeks of age	
*Eμ-Myc* HPCs expressing AKT, Pim2, or vector inoculated into lethally irradiated syngeneic wild-type recipients	*Eμ-Myc*: HPCs with vector; pre-B-cell lymphomas with 20% incidence at 14 weeks *Eμ-Myc; Arf* ^−^*^/^*^−^ tumors responded to doxorubicin or rapamycin but responded to the combination	*Eμ-Myc* HPCs with *Pim2*: pre-B-cell lymphomas with 100% incidence at 10.7 weeks of age. Resistant to doxorubicin, rapamycin, and combination treatment
		*Eμ-Myc* HPCs with *AKT*: pre-B-cell lymphomas with 100% incidence at 12 weeks. Resistant to doxorubicin and rapamycin but sensitive to combination treatment (63)
*Eμ-Myc: Tsc2*^−^*^/^*^−^ HPCs expressing Pim2 or vector inoculated into lethally irradiated, syngeneic wild-type recipients	*Eμ-Myc: Tsc2*^−^*^/^*^−^ tumors treated with rapamycin relapse-free up to 3 weeks	*Eμ-Myc: Tsc2*^−/−^ with Pim2 tumors treated with rapamycin resistant or relapsed free up to 2 weeks, treatment with silvestrol delayed relapse to 2.7 weeks (63)
*pim1;PSA61-Cre; pten^+/^*^−^	Prostate and bladder epithelium	Hormone-induced high-grade mPIN lesions in cooperation with *pten* loss; no cooperation in aging-induced mPIN. Increased inflammation surrounding target tissues leading to pyelonephritis with 100% penetrance in 16-week-old mice (33)
		One round of hormone treatment induced high-grade bladder hyperplasia in cooperation with *pten* loss
		86% Penetrance in 16 weeks old mice (34)

## Pim Transgenic Models and Carcinogens

Generally, the spontaneous tumor rate in Pim1 transgenic mice is low. As has been described above, only 10% of *Eμ-pim1* mice develop spontaneous T-cell lymphoma after 240 days. These tumor rates drastically increase after treatment with carcinogens. Breuer et al. showed that treatment with a single low dose of *N*-ethyl-*N*-nitrosourea (ENU) increases the incidence of tumors to 100% within 200 days. c-myc overexpression could be detected in all of the ENU-induced lymphomas, underlining the synergistic effects of both genes (23). Further studies also showed a high dose-dependency of tumor incidence and c-myc overexpression to ENU treatment (76). Due to these results, it was estimated that *Eμ-pim1* transgenic mice were 25-fold more susceptible to ENU-induced carcinogenesis than non-transgenic littermates (76).

The synergistic effect of c-myc and pim1 in tumor development was also shown in methyl methanesulfonate (MMS)-treated AKR mice, where MMS accelerated tumor development. These tumors do not show K-*ras* mutations, but they do show integration in the c-*myc* and *pim-1* loci by endogenous murine leukemia virus, most likely due to the insertion of proviral DNA (77). When AKR mice were treated with *N*-methyl-*N*-nitrosourea (MNU), tumor development started as early as 3 months of age, and *pim1* and *c-myc* gene rearrangements due to proviral integration were observed in several of the resulting thymomas. Spontaneous tumors in AKR mice (which usually develop at 6 month of age) are distinct, and the development of thymomas that contain proviral integrations at the *pim1* locus in the MNU-treated AKR mice involves the cooperation between the chemical carcinogen and endogenous murine leukemia virus (78).

Because the mortality in Pim1 mice associated with ENU exposure was closely related to the onset of neoplastic development (79), the Pim1 transgenic mouse models were considered optimal to test carcinogens and to assay agents for the chemoprevention of lymphomas.

McCormick and colleagues assessed the efficacy of *N*-(4-hydroxypheny)-all-trans-retinamide (4-HPR) and α-difluorometh ylornithine (DFMO) as chemopreventive agents in the Pim-1 lymphoma model system treated with ENU. Although DFMO had no effect on lymphoma incidence, latency, or mortality, a high dose of 4-HPR greatly reduced both lymphoma incidence and mortality at 20 weeks post-ENU administration; at 20 weeks post-ENU, the incidence of lymphoma and the mortality rate of animals fed with high dose of 4-HPR were half of those of the dietary control cohorts. However, upon termination of the study (at 35 weeks), the lymphoma incidence and mortality were similar across all study groups, implying that the chemoprevention provided by 4-HPR only represents a delay rather than an inhibition of tumor development (79).

Because *Eμ-pim1* mice are 25-fold more susceptible to ENU-induced lymphoma, this mouse model seemed to be useful to test a variety of other carcinogens. For example, 2-amino-1-methyl-6-phenylimidazo[4,5-b]pyridine (PhIP), a potent mouse lymphomagen, and 2-amino-3-methylimidazo[4,5-/]quinoline (IQ), a liver carcinogen that also causes lung tumors and tumors of the forestomach in mice, were tested in *Eμ-pim1* mice. Both carcinogens were included into the standard diet at 0.03%. Whereas transgenic *Eμ-pim1* mice are highly susceptible to PhIP-induced lymphomagenesis, they do not respond to the IQ treatment. PhIP increased the number of tumors and reduced the latency of tumor development; surprisingly, this effect was strongest in female *Eμ-Pim1* mice (80, 81).

A different study also tested four genotoxic procarcinogens in the *Eμ-Pim1* transgenic model: 2-acetylaminofluorene (AAF), *n*-nitrosodiethylamine (NDEA), 1,2-dichloroethane (1,2-DCE), and benzene (BEN). These compounds all require metabolic activation and, with the exception of benzene, are not mouse lymphomagens (82). Over the time course of 38 weeks, the compounds were administered daily by oral gavage. Only a small, but statistically significant, increase in the incidence of malignant lymphomas was observed in the males treated with a high dose of AAF, females treated with high and low doses of NDEA, and females treated with a high dose of DCE (82). However, BEN, the only carcinogen known to be a lymphomagen, did not increase the incidence of tumors significantly. The authors here argued that the study time for BEN might have been too short because other studies of BEN-induced lymphoma detected tumors after 48 weeks in female mice and 54 weeks in male mice.

The study presented by Kroese and colleagues tested two chemicals that target the lymphohematopoietic system, Benzo[a]pyrene (B[a]P) and 12-*O*-tetradecanoylphorbol-13-acetate (TPA), in the same *Eμ-pim-1* mouse model. B[a]P, given three times a week by oral gavage for 13 weeks at 4.3, 13, or 39 mg/kg body weight resulted in a dose-dependent increase in lymphomas, with up to a 90% incidence in *Eμ-Pim1* mice during the observation period of 40 weeks; in contrast, the non-transgenic mice did not develop lymphomas. B[a]P also induced tumors of the forestomach within this observation period, although at a lower incidence and with an apparently equal effectiveness in the wild-type (83). However, in this study, TPA did not induce lymphoma or other tumor types in any tested genotype. The authors speculated that the negative results for TPA were due to unresponsiveness to non-genotoxic chemicals that only provide tumor-promoting activity (83).

Another study showed that mitomycin c does not act as a lymphomagenic agent in *Eμ-pim1* mice (84). It was also shown that *Eμ-Pim1* mice were at least threefold more sensitive to fractionated total body X-irradiation than non-transgenic control mice. After X-irradiation, transgenic mice developed T-cell lymphomas with high levels of c-myc in 75% of the tumors and a three to fivefold increase in endogenous pim1 (85, 86). Lymphoma also developed in the transgenic mice with a shorter latency than in wild-type littermates. This assay showed that *Eμ-Pim1* mice are highly susceptible to X-ray-induced lymphoma and thus susceptible to carcinogens that act directly to induce large chromosomal deletions and rearrangements rather than point mutations (84).

Altogether, the *Eμ-Pim1* mouse model is a highly sensitive *in vivo* system for short-term carcinogen testing that is limited to genotoxic carcinogens that induce lymphomagenesis through large gene deletions and rearrangements.

Most carcinogenic assays elucidating pim kinase function were carried out in Pim1 transgenic mice, but similarities can be observed when using transgenic mice that overexpress human Pim-3 in the liver. These mice do not develop HCC spontaneously, but when treated with DEN, Pim-3 transgenic mice develop a significantly higher incidence of HCC than treated wild-type mice. This might indicate that other Pim transgenic mouse models are also suitable for short-term assays using carcinogens (35).

The increased susceptibility of Pim mouse models to carcinogens is summarized in Table 6.

**Table 6:** **Carcinogens strongly induce tumorigenesis in Pim mouse models**.

Mouse model and phenotype	Carcinogen: dose and time of treatment	Carcinogen action	Phenotype	Increased levels of oncogenes or K-ras mutation
*Eμ-pim1*: 10% mice developed T-cell lymphoma, at 34 weeks	ENU 200, 60, 15, 4.1, or 0.1 mg/kg, 15 days after birth	Small alkyl DNA adducts	T-cell lymphomas: latency of 17 weeks for the highest dose and 34 weeks for the lowest doses Penetrance: 100–70% for the three highest doses and 20% for 4–0.1 mg 7 kg dose	Enhanced expression of c-myc without rearrangement or amplifications. Incidence of mutations: 4.5% *N-ras* mutations, 9% *K-ras* mutations (23, 76)
	ENU 50 mg/kg in combination with dietary administration of 4-HPR, DFMO as chemopreventive agents	Small alkyl DNA adducts	Infiltrative metastatic lymphomas. Only 4-HPR induces a dose-related delay in tumor progression	n.d. (79)
	Diet of 0.03% PhiP for 7 months or 0.03% IQ for 6 months	Carcinogenic or mutagenic, respectively	Lymphoblastic lymphoma in 80% of the females and 27% of the males, 28.5 weeks after treatment	n.d. (80, 81)
	25 and 100 mg/kg AAF, 1 and 3 mg/kg NDEA, 100 and 300 mg/kg 1,2-DCE, and 50 and 100 mg/kg BEN	Genotoxic procarcinogens	Pleomorphic, lymphocytic lymphomas, and leukemias. Small but significant increase in incidence of malignant lymphomas in males treated with a high dose of 2-AAF (3.4-fold), the females treated with high and low doses of NDEA (2.5-fold), and females treated with a high dose of 1,2-DCE (1.8-fold)	n.d. (82)
	4.3, 13, or 39 mg/kg oral administration of B[a]P for 13 weeks and topical administration of 10 μg TPA twice a week; 7 weeks interruption and 35 weeks of 3 μg treatment. 220 μg total TPA dose/mouse	B[a]P requires metabolism and generates bulky DNA adduct. TPA is a tumor promoter	Multicentric lymphoma and T-cell lymphomas with B[a]P B[a]P dose-dependent induction of lymphomas in males, starting at 25 days. Transgenic mice five time more sensitive that wild-type counterparts	75% Of mice had increased *c-myc* levels, and 12.5% of tumors harbored *K-ras* mutations (83)
	Total body X-irradiation	DNA strand breaks	Dose-dependent incidence of T-cell lymphomas: (4 × 1.5 Gy X-ray: 100%; 4 × 1.0 Gy X-ray: 90%; 4 × 0.5 Gy X-ray: 28%) 36 weeks after the last dose	75% Of mice had 5–20- fold expression of *c-myc*, and 16% mice have increased pim-1 levels (85, 86)
	Mitomycin c, cumulative dose of 2.67–6.55 mg/kg	DNA cross-linking agent	11–30% Of females developed T-cell lymphomas with no dose response effect	n.d. (84)
*alb-pim3*	DEN 10 mg/kg	Liver injury	81% Males developed hepatocellular carcinoma after DEN treatment for 10 months (versus 41% of wild-type mice)	n.d. (35)
AKR mice: spontaneous thymomas at >24 weeks old. MCF provirus integration: 17% *pim1*, 6.66% c-*myc*	NMU 50 mg/kg, 4-week-old mice	Methylating agent; G-A transition mutations	Thymomas between 12 and 24 weeks	Ecotropic-like provirus integration; 3.84% *c-myc* and 9.33% *pim-1*. 24% *K-ras* mutations (78)
	MMS 120 mg/kg, 4-week-old mice	Methylating agent: weak mutagen	Thymomas developed between 20 and 50 weeks in 100% of mice	MCF (mink cytopathic focus-forming) provirus integration 19.2% *c-myc* and 4% *pim-1*; no mutations in *K-ras* (77)

*n.d., not determined*.

## Conclusion

Pim1 was discovered as a proviral insertion site in Moloney-murine leukemia virus in T-cell lymphomas, and further studies identified the other two family members, pim2 and pim3, as alternative integration sites. This discovery prompted the development of transgenic mouse models of pim1 to study its oncogenic behavior. The *Eμ-pim1* mouse model has been broadly used to study oncogenic cooperation by infection with M-MuLV and by crosses with other transgenic models; this model has also been extensively used for carcinogen testing. *pim-1* is a weak oncogene that shows strong synergy with *c-myc* in lymphomas and prostate tumors. Double-transgenic mouse models confirmed the cooperation between most of the complementation genes found by the infection of Pim transgenic mice with M-MLuV.

The development of pim isoform-specific KO mice and double and triple KO mice helped to understand the contribution of each isoform to hematopoiesis as well as to sarcoma development and progression.

However, there is still much work that must be performed to fully understand the contributions of pim2 and pim3 to tumorigenesis and to elucidate the effects of the pim family on the complex signaling network in which they are imbedded.

## Conflict of Interest Statement

The authors declare that the research was conducted in the absence of any commercial or financial relationships that could be construed as a potential conflict of interest.
